# The lncRNA CCAT1 upregulates TGFβR1 via sponging miR-490-3p to promote TGFβ1-induced EMT of ovarian cancer cells

**DOI:** 10.1186/s12935-018-0604-1

**Published:** 2018-09-20

**Authors:** Yang Mu, Na Li, Yu-Lan Cui

**Affiliations:** Department of Gynaecology, The Second Affiliated Hospital of Harbin Medical University, No. 146, Baojian Road, Harbin, 150086 Heilongjiang People’s Republic of China

**Keywords:** Ovarian cancer, EMT, *CCAT1*, *miR*-*490*-*3p*, TGFβ1/TGFβR1

## Abstract

**Background:**

Ovarian cancer is the fifth leading cause of cancer deaths in women worldwide. *LncRNACCAT1* was reported to play a critical role in cell metastasis of ovarian cancer. However, little is known about the detailed mechanism of how *CCAT1* enhances TGFβ1-induced EMT of ovarian cancer cells.

**Methods:**

We used RT-qPCR to examine the level of *miR*-*490*-*3p* and *CCAT1* and western blot to detect the protein level of TGFβR1 and EMT-associated markers. We utilized luciferase reporter assay to confirm the direct interaction of *CCAT1* or TGFβ1 with *miR*-*490*-*3p*. Wound healing and invasion assay were employed to investigate the role of *CCAT1* and *miR*-*490*-*3p* in the TGFβ1-induced migration and cell invasion of ovarian cancer cells, respectively.

**Results:**

TGFβ1 stimulated the expression of CCAT1. And CC*AT1* knockdown decreased cell migration, invasion and EMT-associated markers expression of ovarian cancer cells treated with TGFβ1. *CCAT1* directly targeted and downregulated *miR*-*490*-*3p*, then increasing TGFβR1 level. *miR*-*490*-*3p* was shown to regulate cell invasion, migration and EMT markers expression via TGFβR1. In addition, we also observed that *miR*-*490*-*3p* was essential for TGFβ1-induced tumor cell invasion and migration influenced by *CCAT1*. *CCAT1* level was significantly higher in tumors than adjacent normal tissue, in contrast, *miR*-*490*-*3p* level was lower in ovarian tumors.

**Conclusion:**

Here, we reveal that *CCAT1* contributes to TGFβ1-induced EMT of ovarian tumor cells through *miR*-*490*-*3p*/TGFR1 axis. These findings will provide deep insights into the mechanism by which *CCAT1* exerts its oncogenic role in ovarian cancer progression and facilitate developing novel therapeutical therapies for treating ovarian cancer.

## Background

Ovarian cancer is one of the most lethal cancers and the fifth leading cause of cancer-associated death. However, little improvement of survival rate has been achieved over the past decade [1–3]. Patients diagnosed and treated with early stages have a 5-year survival rate over 90%. Unfortunately, the vast majority of ovarian cancer patients are diagnosed with advanced disease and 5-year survival is less than 30% [4]. Hence, the comprehensive understanding of the molecular mechanism of ovarian cancer metastasis is a key issue.

Epithelial–mesenchymal transition (EMT) is a developmental process whereby epithelial cells reprogram to a mesenchymal-like phenotype. Tumor cells undergo EMT change, a key prerequisite for metastasis, which can be initiated or controlled by various intracellular signaling pathway in response to environmental cues, including transforming growth factor beta1(TGFβ1) signaling [5, 6]. On one hand, TGFβ1 directly induces expression of EMT transcription factors, such as Snail, Slug, zinc finger E-box-binding homeobox1/2(ZEB1/2) and Twist, through Smad pathway [7, 8]. One the other hand, TGFβ1 promotes EMT via activation of PI3K/Akt/mTOR or mitogen-activated protein kinase (MAPK) pathway [9, 10]. Several studies suggest that the TGFβ1 is involved in ovarian cancer EMT progression. For example, it was report that TGFβ1 was upregulated in ovarian CAF-derived exosomes, which enhanced migration and invasion ability and the promotion of EMT by activating the SMAD signaling pathway [11]. Inhibitor of DNA binding 1 (Id-1), a protein repressed by miR-29b, facilitates the TGFβ1-induced EMT in human ovarian cancer cells [12]. However, little is known about the detailed mechanism of how TGFβ1 induces EMT of ovarian cancer cells.

Long ncRNAs, defined as a form of ncRNAs greater than 200 nt in length, are found to exert their gene transcription regulatory function by epigenetic regulatory mechanism [13–15]. Colon cancer-associated transcript 1 (*CCAT1*), ~ 2-kb lncRNA located at chromosome 8q24.21, is first found to be upregulated in colon cancer [16]. Recently, *CCAT1* has been reported to be involved in a variety of cancers, including hepatocellular carcinoma [17], gallbladder cancer [18], gastric cancer [19] and colorectal cancer [20]. Yuan Cao et al. showed that *CCAT1* downregulation inhibited epithelial ovarian cancer cell EMT, migration and invasion through targeting miR-152 and miR-130b [21]. However, whether CCAT1 is implicated in TGFβ1-induced EMT of ovarian tumor cells remains unclear. Based on the above facts, we sought to clarify the mechanism by which CCAT1 promoted TGFβ1-induced EMT of ovarian cancer cells.

Over the past decades, microRNAs have been considered to modulate their target genes expression by binding the 3′-UTR of targeted genes. Pathologically, microRNAs are involved in a wide range of cancer cell phenotypes, such as cell proliferation, survival, invasion and EMT [22, 23]. For examples, aberrant expression of miR-200 family is strongly associated with pathologic EMT [24]. MiR-451 regulates migration of glioma cells through AMPK and mTOR signaling [25]. In bladder cancer, miR-148a suppresses EMT by establishing links between ERBB3/AKT2/c-myc and DNMT1 [26]. Recently, several studies have showed that *miR*-*490*-*3p* has an inhibitory role in EMT of hepatocellular carcinoma and colorectal cancer cells [27, 28]. Intriguingly, *miR*-*490*-*3p* inhibits colorectal cancer metastasis by targeting TGFβR1, a TGFβ1 cognate receptor [29]. Moreover, it was report that lncRNACCAT1 regulated gastric cancer cell migration by targeting *miR*-*490*-*3p* [30]. Besides, *MiR*-*490*-*3p* plays a tumour suppressor role in epithelial ovarian cancer,and overexpression of *miR*-*490*-*3p* was reported to promote G1/S arrest and apoptosis, reduce cell proliferation and invasion of ovarian cancer cells [31]. It remains unknown about whether CCAT1 regulates TGFβ1-induced EMT of ovarian tumor cells through *miR490*-*3p*.

In this study, we highlight that knockdown *CCAT1* represses TGFβ1-induced EMT of ovarian cancer cells through *miR*-*490*-*3p*/TGFβR1 axis. These findings will provide more understanding of how *CCAT1* contributes to ovarian cancer metastasis, which helps develop novel targeted drugs for treating ovarian cancer.

## Materials and methods

### Cell culture and transfection

Ovarian cancer cells (SKOV3 and CaOV3) and 293T cell were purchased from ATCC and cultured in Dulbecco’s Modified Eagle’s Medium (DMEM, Hyclone) supplemented with 10% fetal bovine serum and 100 U/ml penicillin/streptomycin at 37 °C, 5% CO_2_. TGFβ1 was purchased from R and D systems and was used to induce EMT in SKOV3 and CaOV3 (10 ng/ml) cells for the indicated time periods.

The TGFβR1 cDNA was subcloned into pCDNA3.1 vector which was transfected into cells using lipofectamine 2000 according to the instruction. For *miR*-*490*-*3p* mimics or *miR*-*490*-*3p* inhibitor transfection, we used LipofectamineVR LTX with PlusTM Reagent (Life Technologies) to transfect them into cells. All siRNAs, *miR*-*490*-*3p* mimics and *miR*-*490*-*3p* inhibitor were synthesized by GenePharma. The sequences are as follows:

*miR*-*490*-*3p* mimics: (sense) 5′-CAACCUGGAGGACUCCAUGCUC-3′; (antisense) 5′-GCAUGGAGUCCUCCAGGUUGUU-3′;

*miR*-*490*-*3p* inhibitor: 5′-CAGCAUGGAGUCCUCCAGGUUG-3′.

### Patients and samples

A cohort of 25 ovarian tumor tissues and adjacent normal ovarian tissue samples were obtained from patients aged 25–55 undergoing wedge biopsy of the ovaries or adnexectomy due to myoma or adenomyosis, between 2016.6 and 2017.5. No patients had received chemotherapy or radiotherapy prior to surgery. Consent from all patients were obtained. Ovarian cancer was validated by histological examination in all cases according to World Health Organization criteria. Ovarian cancer and normal ovarian tissue specimens excised surgically from patients were immediately snap-frozen and stored in liquid nitrogen until use. This experiment was approved by ethic committee of the 2nd Affiliated Hospital of Harbin Medical University, and the tissues were acquired with the consent of patients.

### Plasmid transfection and lentivirus package

The short hairpin RNAs (*shRNA CCAT1*) were cloned into PLKO.1 vector. To make lentiviruses, the packaging vectors (pPAX2 and pVSVG) and PLKO. shRNAs were co-transfected into 293T cells. The supernatant was harvested at 48 h after transfection. For virus infection, the virus supernatant was added to medium at 1:5 ratio, after 24 h, 2 μg/ml puromycin was used to select the positive cells.

### Wound healing assay

Migration of cells were measured by a wound healing assay in vitro. Briefly, 2 × 10^5^ SKOV3 or CaOV3 cells were seeded onto 6-well plates, with either sh-*CCAT1* or sh-NC, and incubated in appropriate complete culture medium for 16 h under normoxic conditions at 37 °C. The monolayer was scratched and incubated in medium without FBS for 24 h. The wound width was measured after 24 h. Three different locations were visualized and photographed under inverted microscope.

### Invasion assay

Invasion assay was performed using chambers with 8.0-μm pore membranes (Millipore). Ovarian cancer cells (1 × 10^5^ cells) were resuspended in 200 µl of FBS-free medium, and then seeded into the top chamber with Matrigel-coated membrane. Next, 500 µl medium with 10% FBS was added to the bottom chamber as a chemoattractant. After 48 h of incubation, the invaded cells were fixed, stained with 0.005% crystal violet, and counted under the inverted microscope.

### Luciferase reporter assays

*CCAT1* or TGFβR1 mutant was generated using site-directed mutagenesis. Then, the sequence of the *CCAT1* or TGFβR1 was cloned into the firefly luciferase-expressing vector pGL3-luciferase plasmid. As for luciferase assay, the SKOV3 or CaOV3 cells were seeded for triplicates in 24-well plates at the day before transfection, and co-transfected with the *CCAT1* or TGFβR1 reporter vector and *miR*-*490*-*3p*. Then, the cells were harvested and lysed, and the luciferase activities were assayed using the Dual-Luciferase Reporter System (Promega). Three independent experiments were performed.

### Western blot

The cells were harvested and washed with PBS buffer, then lysed by 1 × SDS loading buffer. The lysates were boiled at 100 °C for 5 min. The samples were centrifuged at 10,000 rpm for 1 min. Around 50 μg of total proteins was loaded onto SDS-PAGE gel and resolved. After that, the proteins were transferred to PVDF membrane at 300 mA for 1.5 h. The membrane was blocked with 5% non-fat milk in 1× TBST for 1 h at room temperature, the membrane was then incubated with primary antibodies at 4 °C overnight. The following day, the membrane was washed with 1× TBST for three times, 5 min each time. The membrane was incubated with secondary antibodies at room temperature for 1 h. Finally, the membrane was incubated with ECL solution and then exposed. The following antibodies were used: anti-TGFβR1 (cell signaling technology, USA), anti-E-cadherin (cell signaling technology, USA), anti-N-cadherin (cell signaling technology, USA), anti-Claudin (cell signaling technology, USA), anti-β-actin (Proteintech, USA), anti-MMP9 (Abcam, USA), anti-GAPDH (Proteintech, USA).

### RT-qPCR

We extracted the RNA using Trizol method. Cells were lysed by Trizol buffer and then add chloroform to the mixture. The sample was centrifuged at 12,000 rpm for 10 min and transferred to new EP tube, mixed with equivalent volume of isopropanol, next, the resultant was centrifuged at 12,000 rpm for 10 min. Removing the supernatant and add 75% ethanol to wash the pellet and centrifuge. Finally, discard the ethanol and dry the pellet, use 20–30 μl Rnase-free H_2_O to resolve the RNA.

For reverse transcription, about 1 μg of total RNA was used for reverse transcription according to manufacturer instruction (TAKARA PrimeScript Kit). The expression of *miR*-*490*-*3p* was quantified by TaqMan miRNA assays (Applied Biosystems, Foster City, CA, USA).

For real time PCR, we used SYBR as probe dye and detected the signal, the GAPDH and U6 were used as internal control. The following primers were used:

*CCAT1*-QPCR-F: 5′-GCAGGCAGAAAGCCGTATCT-3′

*CCAT1*-QPCR-R: 5′-TCCCAGGTCCTAGTCTGCTT-3′

*miR*-*490*-*3p*-QPCR-F: 5′-CGCAACCTGGAGGACTCC-3′

*miR*-*490*-*3p*-QPCR-R: 5′-CGGCCCAGTGTTCAGACTAC-3′

TGFβR1-QPCR-F: 5′-GTGACAGATGGGCTCTGCTT-3′

TGFβR1-QPCR-R: 5′-AGGGCCAGTAGTTGGAAGTT-3′

*Claudin*-QPCR-F: 5′-TTTACTCCTATGCCGGCGAC-3′

*Claudin*-QPCR-R: 5′-GAGGATGCCAACCACCATCA-3′

*E*-*cadherin*-QPCR-F: 5′-TCACATCCTACACTGCCCAG-3;

*E*-*cadherin*-QPCR-R: 5′-AGTGTCCCTGTTCCAGTAGC-3′,

N-cadherin-QPCR-F: 5′-AGGGGACCTTTTCCTCAAGA-3′;

N-cadherin-QPCR-R: 5′-TCAAATGAAACCGGGCTATC-3′,

*Vimentin*-QPCR-F: 5′-GGACCAGCTAACCAACGACA-3′;

*Vimentin*-QPCR-R: 5′-AAGGTCAAGACGTGCCAGAG-3′,

MMP9-QPCR-F: 5′-TTCCAAACCTTTGAGGGCGA-3′;

MMP9-QPCR-R:5′-CTGTACACGCGAGTGAAGGT-3′,

GAPDH-QPCR-F: 5′-AGCCCAAGATGCCCTTCAGT-3′;

GAPDH-QPCR-R: 5′-AGCCCAAGATGCCCTTCAGT-3′,

U6-QPCR-F: 5′-CTCGCTTCGGCAGCACA-3′;

U6-QPCR-R: 5′-AACGCTTCACGAATTTGCGT-3′.

### Statistical analysis

Each experiment was performed for three times, all values were presented as mean ± SD, comparison of two groups were performed using the two-tailed unpaired student’s t-test. One-way ANOVA was used for comparison among multiple groups and multiple comparisons were further performed using post hoc Turkey test. *P < 0.05 were considered statistically significant (*P < 0.05, **P < 0.01, and ***P < 0.001).

## Results

### LncRNA *CCAT1* depletion attenuates TGFβ1-induced EMT of ovarian cancer cells

**Fig. 1:**
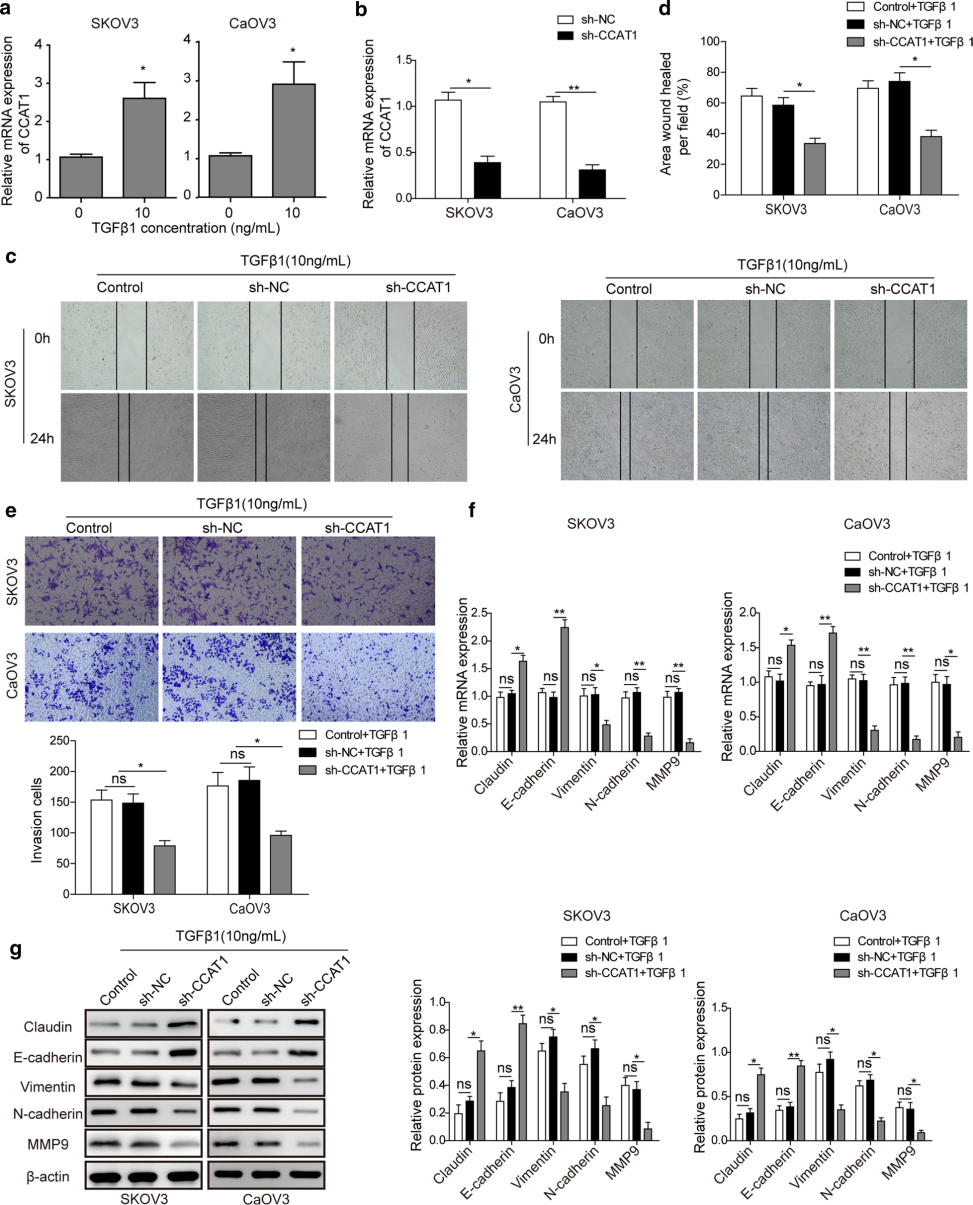
LncRNA CCAT1 depletion attenuates TGFβ1-induced EMT of ovarian cancer cells. **a** The level of CCAT1 in ovarian cancer cells (SKOV3 and CaOV3) was detected by RT-qPCR at 48 h after treated with TGFβ1 (10 ng/ml). **b** RT-qPCR analysis was performed to confirm target gene *CCAT1* silencing after shRNA treatment. RNA level of *CCAT1* in sh-NC (scramble) and sh-*CCAT1* (*CCAT1* knockdown) were detected in ovarian cancer cells (SKOV3 and CaOV3). **c**, **d** Representative images of wound healing assay (**c**) and quantification (**d**) carried out in control (wildtype), sh-NC (scramble) and sh-*CCAT1* (*CCAT1* knockdown) ovarian cancer cells (SKOV3 and CaOV3) treated with 10 ng/ml TGFβ1. **e** Cell invasion ability of control (wildtype), sh-NC (scramble) and sh-*CCAT1* (*CCAT1* knockdown) ovarian cancer cells (SKOV3 and CaOV3) was measured with transwell invasion assay under 10 ng/ml TGFβ1. **f** the mRNA level of EMT-associated markers (claudin, E-cadherin, N-cadherin, vimentin and MMP9) in control (wildtype), sh-NC (scramble) and sh-*CCAT1* (*CCAT1* knockdown) ovarian cancer cells (SKOV3 and CaOV3) was measured by RT-qPCR analysis after cells treated with 10 ng/ml TGFβ1. **g** Western blot analysis showed the protein level of EMT-associated markers (claudin, E-cadherin, N-cadherin, vimentin and MMP9) after cells were transfected with sh-*CCAT1* (*CCAT1* knockdown) in ovarian cancer cells (SKOV3 and CaOV3) compared with the control (wildtype), sh-NC (scramble) groups under with 10 ng/ml TGFβ1. All data were represented as mean ± SD from three biological replicates (*P < 0.05; **P < 0.01)

To characterize the role of *CCAT1* in TGFβ1-induced EMT of ovarian cancer cells, we first determined the expression level of CCAT1 in SKOV3 and CaOV3 cells when treated with 10 ng/ml TGFβ1 for 48 h. The result showed CCAT1 was upregulated by TGFβ1 (Fig. 1a). Next, we generated stable *CCAT1*-depleted SKOV3 and CaOV3 cells by shRNAs approach (Fig. 1b). And then we employed wound healing method to examine the migration of *CCAT1*-depleted ovarian cancer cells SKOV3 and CaOV3 in the presence of TGFβ1(10 ng/ml). The results showed that scramble (sh-NC) cells exhibited no migration difference compared to control, whereas *CCAT1* knockdown (sh-*CCAT1*) cell significantly compromised migration (reduced by ~ 40–50%) of ovarian cancer cells induced by TGFβ1 in relative to sh-NC group (Fig. 1c, d). In addition, we sought to determine whether TGFβ1-induced invasion of ovarian cells were regulated by *CCAT1* loss. Transwell matrix penetration assay demonstrated that knockdown of *CCAT1* reduced the number of invasive ovarian cancer cells (by ~ 50%) in the presence of TGFβ1 compared to sh-NC group (Fig. 1e). To further investigate the mechanism underlying TGFβ1-induced invasion and migration of ovarian cancer cells regulated by *CCAT1*, we examined the expression of EMT-associated genes in control and *sh*-*CCAT1* ovarian cells. RT-qPCR and western blot analyses showed that *CCAT1* knockdown markedly attenuated TGFβ1-induced expression of vimentin, N-cadherin and MMP9, on the other hand, *CCAT1* knockdown enhanced TGFβ1-induced expression of E-cadherin and Claudin (Fig. 1f, g). Taken together, these results revealed that *CCAT1* loss led to remarkably attenuated EMT of ovarian cancer cells treated with TGFβ1.

### LncRNA *CCAT1* depletion decreases expression level of TGFβR1

**Fig. 2:**
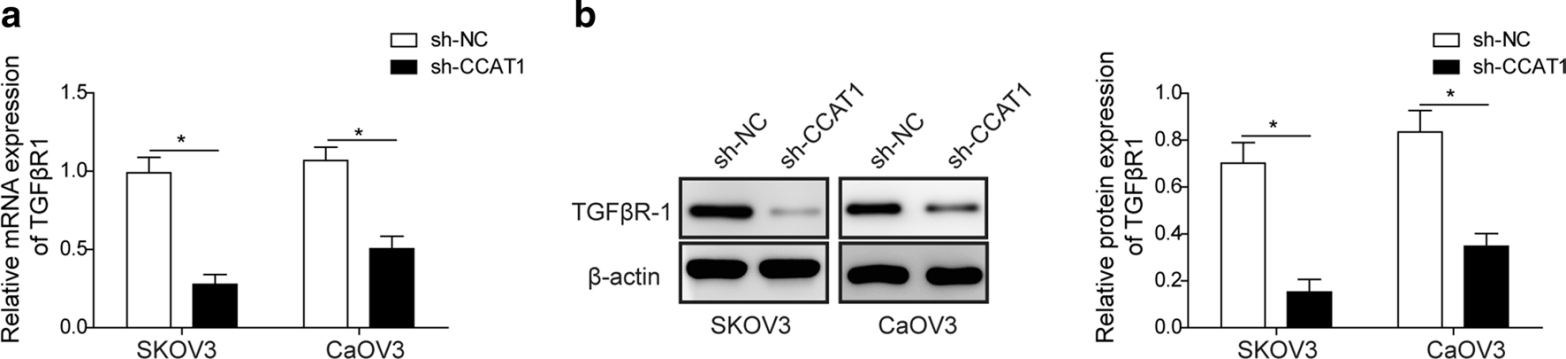
lncRNA *CCAT1* depletion decreases expression level of TGFβR1. **a** RT-qPCR analysis showed the mRNA level of TGFβR1 in sh-NC (scramble) and sh-*CCAT1* (*CCAT1* knockdown) ovarian cancer cells (SKOV3 and CaOV3). **b** The protein level of TGFβR1 in sh-NC (scramble) and sh-*CCAT1* (*CCAT1* knockdown) ovarian cancer cells (SKOV3 and CaOV3) was detected by western blot. All data were represented as mean ± SD from three biological replicates (*P < 0.05)

In order to clarify the mechanism by which *CCAT1* loss compromised TGFβ1-induced EMT, we hypothesized that TGFβ1 cognate receptor, TGFβR1, might be regulated by *CCAT1*. Interestingly, we observed that TGFβR1 mRNA level was diminished by about 50–70% in *CCAT1*-null cells compared to shNC cells (Fig. 2a). In agreement with this, protein level of TGFβR1 was also downregulated by *CCAT1* depletion in both SKOV3 and CaOV3 cells (Fig. 2b). In sum, these data indicated that *CCAT1* played its roles in TGFβ1-induced EMT of ovarian tumor through enhancing TGFβR1 expression.

### LncRNA *CCAT1* directly targets *miR*-*490*-*3p*

**Fig. 3:**
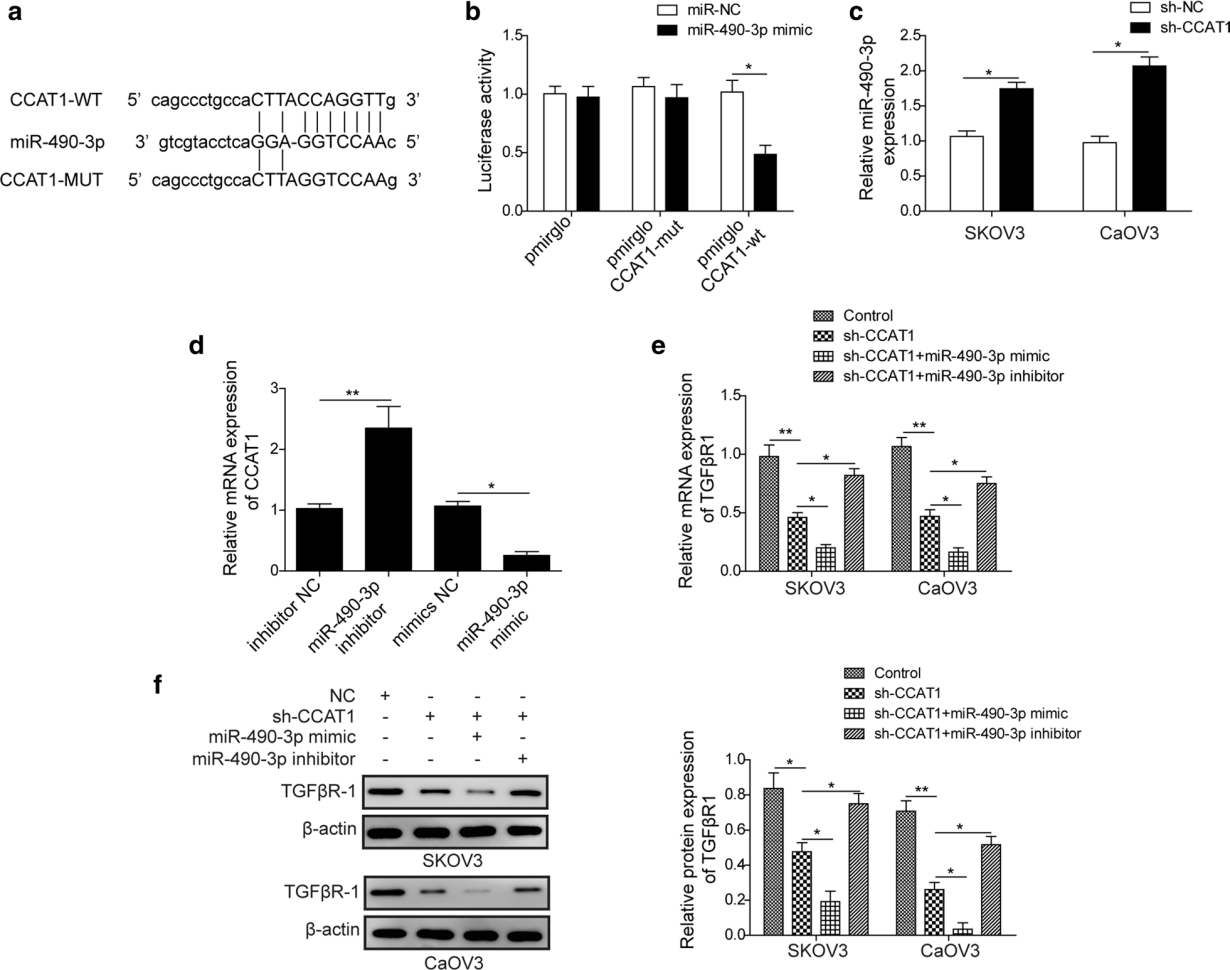
LncRNA *CCAT1* directly targets *miR*-*490*-*3p*. **a** Diagram of bioinformatic prediction of binding site of *miR*-*490*-*3p* by *CCAT1*. **b** Cells were co-transfected with *miR*-*490*-*3p* mimics and wildtype *CCAT1* or mutant *CCAT1*luciferase reporter plasmid. The cell lysates were harvested for luciferase assay. **c** RT-qPCR analysis showed the level of *miR*-*490*-*3p* in sh-NC (scramble) and sh-*CCAT1* (*CCAT1* knockdown) ovarian cancer cells (SKOV3 and CaOV3). **d** The level of CCAT1 of ovarian cancer cells which transfected with *miR*-*490*-*3p* mimic and *miR*-*490*-*3p* inhibitor was detected by RT-qPCR. **e** RT-qPCR analysis showed the mRNA level of TGFβR1 in ovarian cancer cells (SKOV3 and CaOV3) transfected with sh-NC (scramble), sh-*CCAT1* (*CCAT1* knockdown), sh-*CCAT1* (*CCAT1* knockdown) plus *miR*-*490*-*3p* mimics, sh-*CCAT1* (*CCAT1* knockdown) plus *miR*-*490*-*3p* inhibitor. **f** The protein level of TGFβR1 in ovarian cancer cells (SKOV3 and CaOV3) transfected with sh-NC (scramble), sh-*CCAT1* (*CCAT1* knockdown), sh-*CCAT1* (*CCAT1* knockdown) plus *miR*-*490*-*3p* mimics, sh-*CCAT1* (*CCAT1* knockdown) plus *miR*-*490*-*3p* inhibitor was measured by western lot assay. All data were represented as mean ± SD from three biological replicates (*P < 0.05; **P < 0.01)

It has been reported that *miR*-*490*-*3p* has been implicated in ovarian tumor invasion and metastasis [31]. We reasoned that *CCAT1* might exert its role by regulating *miR*-*490*-*3p* in ovarian cancer cells. Bioinformatic analysis revealed that *CCAT1* could directly target *miR*-*490*-*3p* by matching with sequence at 3′-terminus (Fig. 3a). Furthermore, luciferase reporter assay was used to confirm that *CCAT1* directly interacted with *miR*-*490*-*3p*. We found that *miR*-*490*-*3p* overexpression only decreased wildtype *CCAT1*-fused luciferase activity, not *CCAT1* mutant (Fig. 3b). Notably, *miR*-*490*-*3p* level was substantially upregulated by *CCAT1* knockdown in SKOV3 and CaOV3 cells (Fig. 3c). Moreover, miR-490-3p mimics suppressed CCAT1 expression, and instead miR-490-3p inhibitor enhanced CCAT1 expression of SKOV3 and CaOV3 cells (Fig. 3d). RT-qPCR and western blot analyses showed that *miR*-*490*-*3p* overexpression augmented *CCAT1* depletion-induced TGFβR1 downregulation, while *miR*-*490*-*3p* inhibitor greatly restored the expression level of TGFβR1 (Fig. 3e, f). Together, these results suggested that lncRNA *CCAT1* loss downregulated TGFβR1 expression via directly targeting *miR*-*490*-*3p*.

### *miR*-*490*-*3p* is essential for TGFβ1-induced EMT affected by *CCAT1* knockdown via downregulation of TGFβR1

**Fig. 4:**
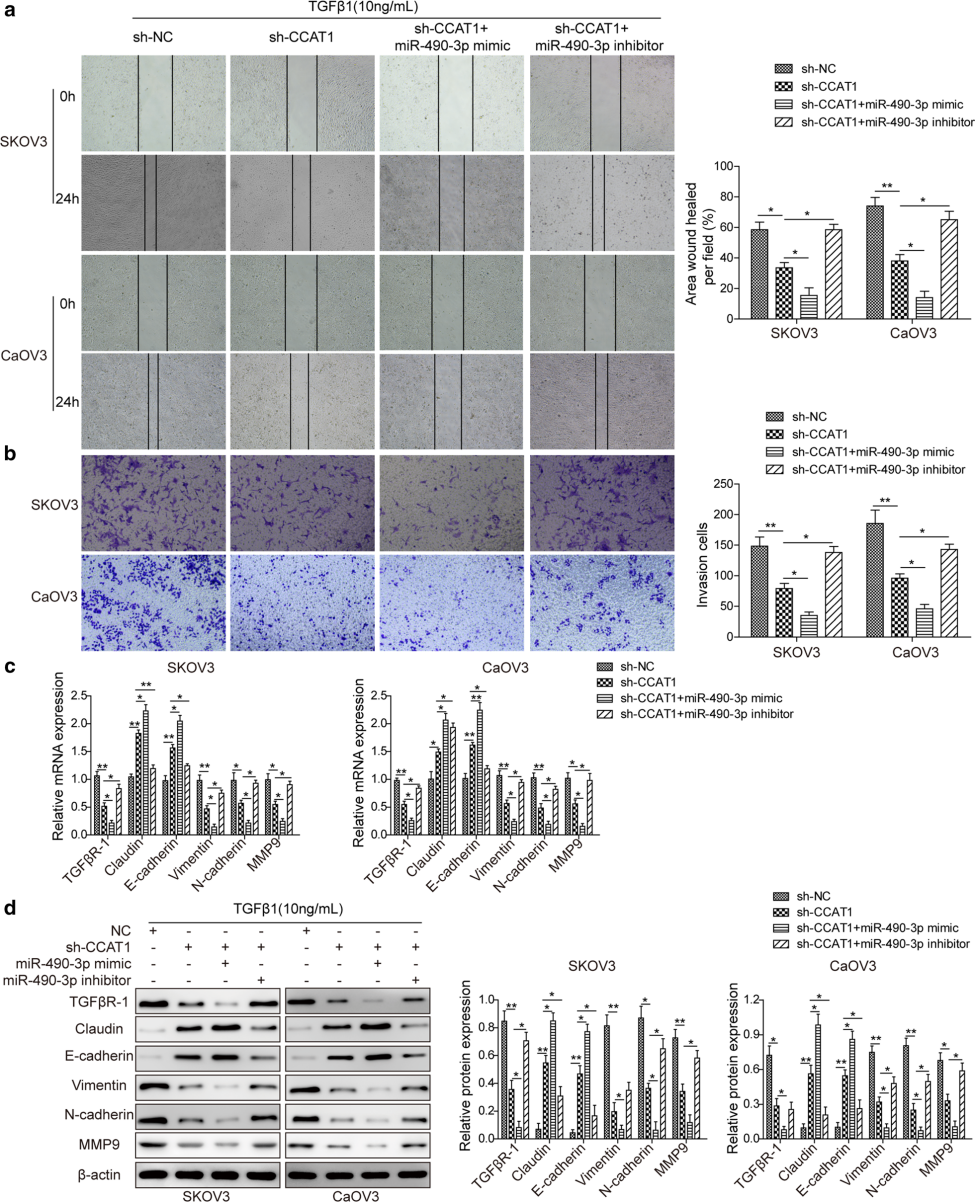
*miR*-*490*-*3p* is essential for TGFβ1-induced EMT affected by *CCAT1* knockdown via downregulation of TGFβR1. **a** Representative images of wound healing assay and quantification carried out in ovarian cancer cells (SKOV3 and CaOV3) transfected with sh-NC (scramble), sh-*CCAT1* (*CCAT1* knockdown), sh-*CCAT1* (*CCAT1* knockdown) plus *miR*-*490*-*3p* mimics, sh-*CCAT1* (*CCAT1* knockdown) plus *miR*-*490*-*3p* inhibitor. All cells were treated with 10 ng/ml TGFβ1. **b** Cell invasion assay and quantification showed invasiveness of ovarian cancer cells (SKOV3 and CaOV3) transfected with sh-NC (scramble), sh-*CCAT1* (*CCAT1* knockdown), sh-*CCAT1* (*CCAT1* knockdown) plus *miR*-*490*-*3p* mimics, sh-*CCAT1* (*CCAT1* knockdown) plus *miR*-*490*-*3p* inhibitor. All cells were treated with 10 ng/ml TGFβ1. **c** The mRNA level of TGFβR1 and EMT-associated markers (claudin, E-cadherin, N-cadherin, vimentin and MMP9) in ovarian cancer cells (SKOV3 and CaOV3) transfected with sh-NC (scramble), sh-*CCAT1* (*CCAT1* knockdown), sh-*CCAT1* (*CCAT1* knockdown) plus *miR*-*490*-*3p* mimics, sh-*CCAT1* (*CCAT1* knockdown) plus *miR*-*490*-*3p* inhibitor was detected by RT-qPCR. All cells were treated with 10 ng/ml TGFβ1. **d** Westernblot analysis showed the protein level of TGFβR1 and EMT-associated markers (claudin, E-cadherin, N-cadherin, vimentin and MMP9) in ovarian cancer cells (SKOV3 and CaOV3) transfected with sh-NC (scramble), sh-*CCAT1* (*CCAT1* knockdown), sh-*CCAT1* (*CCAT1* knockdown) plus *miR*-*490*-*3p* mimics, sh-*CCAT1* (*CCAT1* knockdown) plus *miR*-*490*-*3p* inhibitor. All cells were treated with 10 ng/ml TGFβ1. All data were represented as mean ± SD from three biological replicates (*P < 0.05; **P < 0.01)

As *CCAT1* was shown to decrease *miR*-*490*-*3p* level, then upregulating TGFβR1 expression in SKOV3 and CaOV3 cells; therefore, we sought to assess whether *miR*-*490*-*3p* was essential for *CCAT1*-mediated tumor phenotypes of ovarian cancer cells. Wound healing assay showed that *CCAT1* knockdown alone impaired (decreased by 52%) the ability of TGFβ1 to promote cells migration. Transfection of both *miR*-*490*-*3p* mimics and *CCAT1* shRNA markedly attenuated (dropped by ~ 88%) migration of ovarian cancer cells relative to sh*CCAT1* alone, while co-transfection of *miR*-*490*-*3p* inhibitor and *CCAT1* shRNA into cells exhibited more robust migration (increased by ~ 72%) than sh*CCAT1*-expressing cells (Fig. 4a). Besides, we found that *miR*-*490*-*3p* mimics potentiated sh*CCAT1*-inhibited invasiveness (the invasion cell number decreased by 60%) and instead *miR*-*490*-*3p* inhibitor attenuated (the invasived cell number increased by 61%) sh*CCAT1*-inhibited invasiveness of SKOV3 and CaOV3 cells (Fig. 4b). As for EMT-associated markers, RT-qPCR and westernblot revealed that upregulation of E-cadherin and Claudin by *CCAT1* loss was enhanced by *miR*-*490*-*3p* mimics and attenuated by *miR*-*490*-*3p* inhibitor in SKOV3 and CaOV3 cells, inverse in the expression of vimentin, N-cadherin and MMP9 (Fig. 4c, d). Collectively, these findings indicated that *miR*-*490*-*3p* was essential for TGFβ1-induced EMT of ovarian cancer cells regulated by *CCAT1* depletion.

### *miR*-*490*-*3p* inhibits TGFβ1-induced EMT through directly targeting TGFβR1

**Fig. 5:**
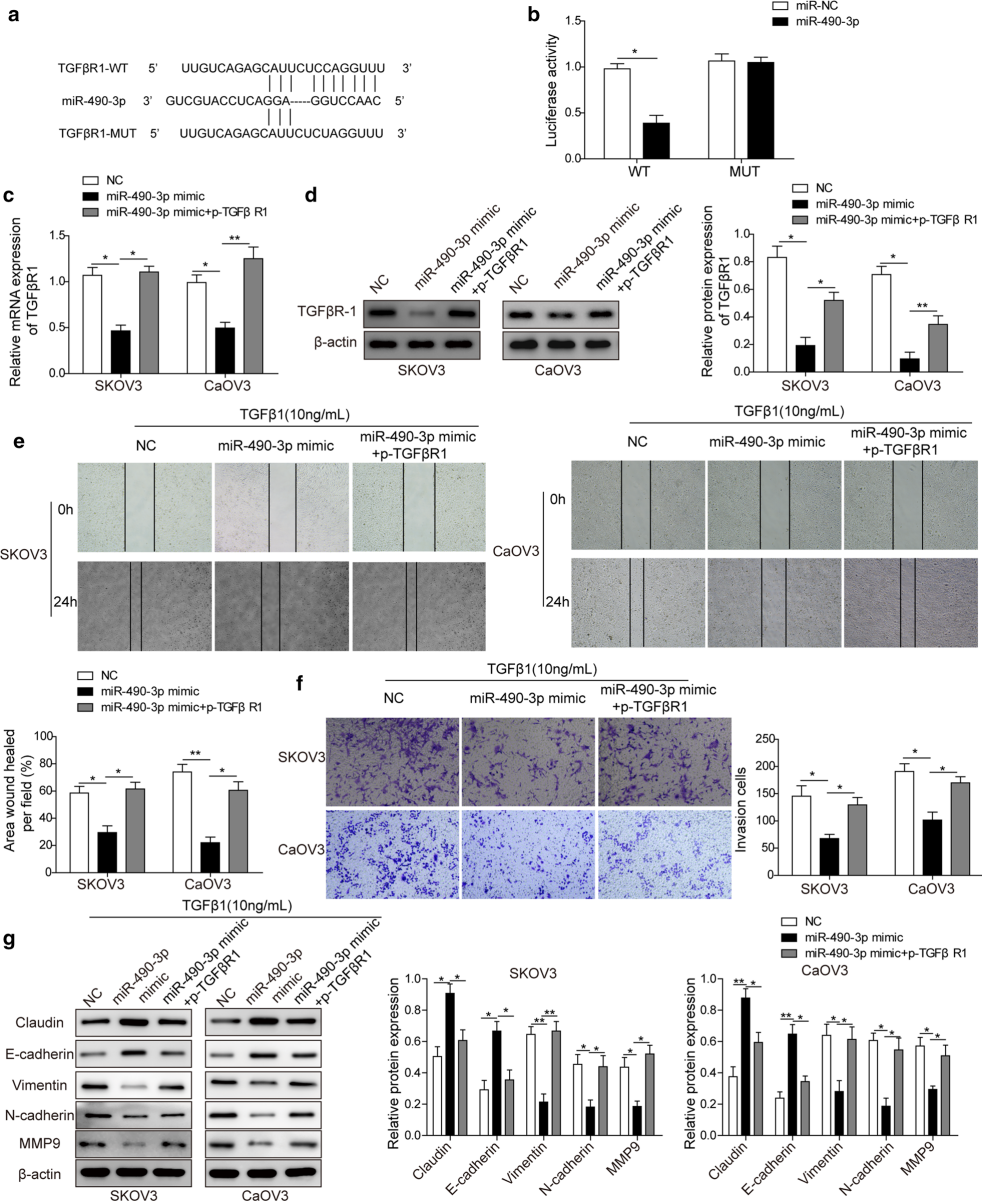
*miR*-*490*-*3p* inhibited TGFβ1-induced EMT through directly targeting TGFβR1. **a** Diagram of bioinformatic prediction of binding site of TGFβR1 by *miR*-*490*-*3p*. **b** Cells were cotransfected with scrambled RNA or *miR*-*490*-*3p* together with TGFβR1-3′-UTR or TGFβR1-mut-3′-UTR luciferase reporter in the presence of firefly luciferase reporter plasmid. Renilla luciferase activity and firefly luciferase activity were measured by dual-luciferase reporter assay. Renilla luciferase activity was normalized to firefly luciferase activity. **c** The mRNA level of TGFβR1 in ovarian cancer cells (SKOV3 and CaOV3) transfected with negative control, *miR*-*490*-*3p* mimics, *miR*-*490*-*3p* mimics plus TGFβR1 was detected by RT-qPCR analysis. **d** Westernblot analysis showed the protein level of TGFβR1 in ovarian cancer cells (SKOV3 and CaOV3) transfected with negative control, *miR*-*490*-*3p* mimics, *miR*-*490*-*3p* mimics plus TGFβR1. β-actin as loading control. **e** Representative images of wound healing assay and quantification carried out in negative control, *miR*-*490*-*3p* mimics or *miR*-*490*-*3p* mimics- plus TGFβR1-overexpressing ovarian cancer cells (SKOV3 and CaOV3) treated with 10 ng/ml TGFβ1. **f** Cell invasion assay and quantification showed invasiveness of negative control, *miR*-*490*-*3p* mimics or *miR*-*490*-*3p* mimics plus TGFβR1 overexpressing ovarian cancer cells (SKOV3 and CaOV3) treated with 10 ng/ml TGFβ1. **g** Westernblot analysis showed the protein level of EMT-associated markers (claudin, E-cadherin, N-cadherin, vimentin and MMP9) in negative control, *miR*-*490*-*3p* mimics or *miR*-*490*-*3p* mimics- plus TGFβR1-overexpressing ovarian cancer cells (SKOV3 and CaOV3). All cells were treated with 10 ng/ml TGFβ1. All data were represented as mean ± SD from three biological replicates (*P < 0.05; **P < 0.01)

To further explore how *miR*-*490*-*3p* affected *CCAT1* loss-induced TGFβR1 expression, we inferred that *miR*-*490*-*3p* probably targeted TGFβR1 and regulated its expression. The bioinformatic analyses revealed that *miR*-*490*-*3p* could target 3′-UTR of TGFβR1 mRNA (Fig. 5a). Luciferase reporter assay demonstrated that *miR*-*490*-*3p* mimics effectively inhibited the activity in wildtype 3′-UTR of TGFβR1 cells, not the mutant, suggesting *miR*-*490*-*3p* played its role via directly binding 3′-UTR of TGFβR1 (Fig. 5b).

To determine the biological function of *miR*-*490*-*3p*-induced TGFβR1 downregulation, we overexpressed *miR*-*490*-*3p* mimics alone or in combination with TGFβR1. The RT-qPCR and westernblot analyses showed TGFβR1 was reduced by *miR*-*490*-*3p* mimics overexpression; however, TGFβR1 level increased when the cells overexpressing *miR*-*490*-*3p* and TGFβR1 (Fig. 5c, d). Next, we observed that *miR*-*490*-*3p* overexpression greatly attenuated migration of ovarian cancer cells SKOV3 and CaOV3, whereas exogenous expression of *miR*-*490*-*3p* and TGFβR1 rescued TGFβ1-induced migration change of the cells (Fig. 5e, f). Similarly, *miR*-*490*-*3p* mimics caused remarkably decreased invasion of ovarian cancer cells compared to negative control, and transfection of *miR*-*490*-*3p* plus TGFβR1 could enhance the invasiveness of ovarian cancer cells comparable to control (Fig. 5f). Finally, we detected EMT-associated markers after overexpression of *miR*-*490*-*3p* and TGFβR1. Our results showed *miR*-*490*-*3p* inhibited TGFβ1-induced expression of vimentin, N-cadherin and MMP9, instead, upregulated TGFβ1-induced expression of E-cadherin and Claudin. More importantly, overexpression of TGFβR1 reverted *miR*-*490*-*3p*-mediated regulation of EMT-related genes in ovarian cancer cells (Fig. 5g).

### LncRNA *CCAT1* negatively correlates with *miR*-*490*-*3p* level in ovarian tumors

**Fig. 6:**
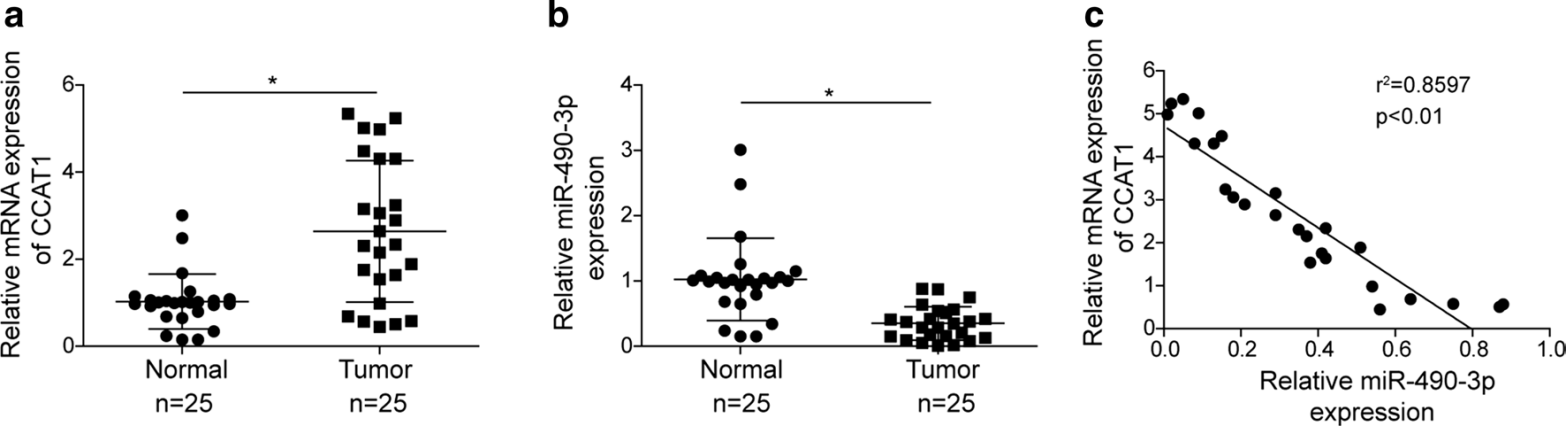
LncRNA *CCAT1* negatively correlates with *miR*-*490*-*3p* level in ovarian tumors. **a** The level of *CCAT1* in ovarian tumors or adjacent tissues (normal) was measured by RT-qPCR, n = 25. **b** The level of *miR*-*490*-*3p* in ovarian tumors or adjacent tissues (normal) was detected by RT-qPCR analysis, n = 25. **c** Pearson’s correlation analysis was used to determine the correlation between the expression levels of *CCAT1* and *miR*-*490*-*3p* in human ovarian tumors; Spearman’s correlation, r^2^ = 0.8597(n = 25). All data were represented as mean ± SD (*P < 0.05)

To determine the clinical association of *CCAT1* and *miR*-*490*-*3p* expression with progression of ovarian cancer, we examined *CCAT1* and *miR*-*490*-*3p* expression level of ovarian tumors (n = 25) and adjacent normal tissues (n = 25) by RT-qPCR approach. *CCAT1* level was higher (~ 2.6 folds) in tumors than in normal tissues; however, *miR*-*490*-*3p* level was lower (~ 65%) in tumors compared to normal tissues (Fig. 6a, b). In addition, the bioinformatic analysis showed negative association between *CCAT1* and *miR*-*490*-*3p* expression in ovarian tumors (r^2^ = 0.8579, p < 0.01) (Fig. 6c). To summarize, our data indicated that CCAT1, clinically, might promote ovarian cancer via inhibiting expression of *miR*-*490*-*3p.*

## Discussion

Ovarian cancer results in the death of about 140,000 women, and limited improvement of survival rate has been achieved in ovarian cancer [1]. Most patients with ovarian cancer died from advanced stage (metastatic) of the cancer, other than early stage [4]. Therefore, it is key to illuminate the mechanism underlying metastasis of ovarian cancer. In this study, our results demonstrated that lncRNA *CCAT1* enhanced TGFβ1-induced metastatic process of ovarian cancer cells via *miR*-*490*-*3p*/TGFβR1 axis, which was crucial for developing targeted drugs for treating ovarian cancer patients with advanced stage.

TGFβ1 signaling is important in a number of cellular processes, physiologically and pathologically [32]. And it is believed that TGFβ1 switches its suppressive role in normal cells into tumor-stimulatory role in cancer cells. Such as, TGFβ1 could induce EMT and metastasis of human ovarian cancer cells [12]. More interestingly, TGFβ1 could modulate EMT by impacting expression of lncRNAs and miRNAs in gastric cancer and bladder cancer. For example, TGFβ1-induced LncRNA UCA1 upregulation promotes gastric cancer invasion and migration [33]. In addition, TGFβ1 secreted by cancer-associated fibroblasts induces EMT of bladder cancer cells through lncRNA-ZEB2NAT [34]. In this study, we first proved that TGFβ1 upregulated expression of lncRNA*CCAT1* in ovarian cancer cells and knockdown of *CCAT1* inhibited TGFβ1-induced EMT. Moreover, consistent with previous studies LncRNA *CCAT1* promotes EMT of intrahepatic cholangiocarcinoma [35]. In addition, it was reported that LncRNA *CCAT1* promoted EMT of epithelial ovarian cancer cells via miR-152/miR-130-Zeb1 axis [21]. All these revealed that TGFβ1 induced EMT of ovarian cancer partly dependent on *lncRNACCAT1*.

Emerging evidence has revealed that lncRNAs exert its effects as competing endogenous RNA (ceRNA) [30]. In the case, lncRNAs commonly interact with miRNAs and mutually regulate each other’s expression. LncRNAs function as ceRNAs to target and degrade miRNAs; however, miRNAs suppress lncRNA through an Argonaute 2-mediated pathway [36, 37]. In the previous report, it was found that CCAT1 is a driver of malignancy, which acts in part through ‘sponging’ miRNA-218-5p in gallbladder cancer [18]. It was also found that C*CAT1* could target and sponge miR-152 in ovarian cancer cells [21]. In this study, we found that *CCAT1* function as ceRNA to directly bind and decline *miR*-*490*-*3p* via complementary sequence. Consistent with the reports that the long noncoding RNA colon cancer-associated transcript-1/miR-490 axis regulates gastric cancer cell migration by targeting hnRNPA1 [30]. *MiR*-*490*-*3p* has been reported to act as oncosuppressive microRNA to inhibit breast cancer tumorigenesis and progression by targeting RhoA directly [38]. Importantly, *miR*-*490*-*3p* may target CDK1 and inhibit ovarian epithelial carcinoma tumorigenesis and progression [31]. Consistent with these results, functionally, we observed that *miR*-*490*-*3p* overexpression led to attenuated migration and invasion, and regulated EMT-associated genes (vimentin, N-cadherin, E-cadherin and Claudin). These data imply that knockdown *CCAT1* inhibited TGFβ1-induced EMT in ovarain cancer cells through sponging *miR*-*490*-*3p*.

Xuehu Xu et al. observed that *miR*-*490*-*3p* targeted TGFβR1 to inhibit colorectal cancer metastasis [29]. Consistently, our results revealed that *miR*-*490*-*3p* suppressed TGFβR1 expression and TGFβR1 overexpression could rescue *miR*-*490*-*3p*-inhibited EMT. J Xiang et al. reported that TGFβR1 promoted EMT of gastric cancer treated with TGFβ, which was attenuated by Grhl2 [39]. Besides, 14-3-3/TGFβR1 axis also promoted tumor metastasis in lung squamous carcinoma [40]. Hence, these conclusions further support our notion described above.

## Conclusion

Here, our results demonstrated that lncRNA *CCAT1* enhanced TGFβ1-induced metastatic process of ovarian cancer cells via *miR*-*490*-*3p*/TGFβR1 axis in ovarian cancer cells. This new molecular axis was confirmed to be important for TGFβ1-induced EMT of ovarian cancer; however, other possible mechanisms responsible for *CCAT1*-mediated metastasis of ovarian cancer cells remains to be investigated for the future. Our findings shed lights on how *CCAT1* regulates TGFβ1-promoted cancer metastasis and facilitate development of effective therapies for treating ovarian cancer.

## Abbreviations

LncRNA: long noncoding RNA
EMT: epithelial–mesenchymal transition
CCAT1: colon cancer-associated transcript 1
TGFβ1: transforming growth factor-β1
TGFβR1: transforming growth factor-β receptor 1
MAPK: mitogen-activated protein kinase
ZEB1/2: zinc finger E-box-binding homeobox1/2
3′-UTR: 3′-untranslated region
DMEM: Dulbecco’s Modified Eagle’s Medium
ATCC: American Tissue Culture Collection
siRNA: short interference RNA
shRNA: short hairpin RNA
MMP9: matrix metalloproteinase9
ceRNA: competing endogenous RNA
RT-qPCR: reverse transcription quantitative real time polymerase chain reaction
